# Digital work tools in occupational health care: A cross-sectional survey of usability and usefulness among occupational health professionals in Finland

**DOI:** 10.1177/20552076261450324

**Published:** 2026-05-12

**Authors:** Sari Nissinen, Pauliina Toivio, Erja Sormunen

**Affiliations:** 1 Finnish Institute of Occupational Health, Occupational Health, Helsinki, Finland

**Keywords:** occupational health, digital tools, customer portals, usability, usefulness

## Abstract

**Objective:**

To examine occupational health care professionals’ experiences of the usability and usefulness of digital work tools (customer portals) and identify factors associated with these perceptions.

**Methods:**

A cross-sectional online survey was conducted among OHC professionals in Finland (n = 95). Usability was assessed using 14 statements based on Nielsen’s dimensions, and usefulness with 15 statements derived from the Technology Acceptance Model. Responses were recorded on a five-point Likert scale. Data analysis included descriptive statistics, correlation analyses, and non-parametric tests to explore associations with background factors.

**Results:**

Usability was generally rated positively, particularly in terms of ease of use, clarity of information presentation and data security. Usefulness was considered highest for supporting collaboration and speeding up service delivery, but the portals do not appear to sufficiently support obtaining an overall picture of work ability and working conditions, nor the monitoring of agreed measures. Good user skills and satisfaction with use were strongly associated with higher ratings (p < 0.001).

**Conclusions:**

Customer portals support individual-level collaboration effectively but require development to strengthen workplace-level information management. Enhancing functionalities and addressing user needs across professional groups are essential for improving the customer portals’ role in occupational health care professionals.

## Introduction

The utilisation of digitalisation has been a strategic goal in social and health care in Finland, accelerated particularly by the COVID-19 pandemic.^1,2^ Thus, digitalisation has transformed the service environment of health care.^3^ Professionals expect technological solutions to improve the quality of work and information exchange with the customer, but if systems are not user-friendly or do not integrate well into service processes, they may instead cause frustration and resistance.^4–6^

Digitalisation is also evident in occupational health care (OHC), where technological solutions have been developed to support collaboration with customers.^3,7,8^ Digital work tools for professionals not only support customer work but also change working practices.^8,9^ According to the sociotechnical model, these effects arise from the interaction of several interconnected factors such as technical solutions, users, work processes and organisational structures.^10^

Customer work in OHC is planned, goal-oriented and based on workplace needs.^11^ Its purpose is to prevent work-related illnesses and accidents and to promote health, work ability and safety.^12^ In Finland, approximately 91% of employees are covered by OHC.^13^ Services such as health examinations and occupational health (OH) counselling are provided through multidisciplinary collaboration between OH physicians, OH nurses, OH psychologists and OH physiotherapists. Customers also include workplaces, for which workplace surveys and advice on improving working conditions are provided. Activities are always planned together with the workplace.^11^

Using digital work tools in customer work requires not only technical competence but also the ability to maintain interaction with the customer in a digital environment.^14^ These tools are most effective when integrated into professionals’ work processes, as isolated solutions may weaken coordination and increase workload.^15–19^ Implementation emphasises orientation, communication of the tool’s purpose and usefulness and easy access to user support, as these influence intentions to use.^15,20^ However, adoption of digital work tools often occurs under time pressure and learning takes place alongside work.^14^

International research shows that digital systems may generate information that is lengthy, repetitive or automatically imported, making it harder for professionals to identify relevant content and increasing cognitive workload^21–23^ This pattern has been described in the digital health literature as contributing to note bloat and a broader administrative documentation burden, where duplicated or auto-imported text obscures essential information and slows down clinical work.^21–23^

Studies on other digital tools used by health professionals highlight that good usability does not automatically translate into practical usefulness. For example, evidence from systematic and scoping reviews of clinical decision support systems shows that although these systems may be easy to operate, they often provide limited support for professionals’ daily work. Particularly when systems do not align with existing workflows or offer help in performing core tasks. This gap between usability and actual usefulness has been repeatedly identified in international digital health research and represents a broader challenge shared across many types of professional digital tools in clinical practice.^24–26^

In this study, digital work tools refer to customer portals used by professionals for communication and document management with workplaces. They also include reporting, monitoring and alert systems as well as data collection and analysis systems related to workplace surveys, which are used to monitor employees’ health and work ability, assess risk, workload and resource factors and prepare recommendations for improving working conditions.^3,4^

Previous research on digital work tools has mainly focused on other health care sectors and patients’ experiences of customer portals.^16,27^ In OHC, Valkonen et al.^28^ have examined older customers’ views on portal use and its opportunities for managing their own health. Users particularly valued timely access to information and the ability to influence. A survey by Nissinen et al.^29^ showed that OHC customers generally consider portals easy to use and supportive of collaboration but obtaining an overall picture of work ability and working conditions is perceived as insufficient.

In addition, research involving OHC professionals has concentrated mainly on the use of telehealth and other remote services.^30,31^ These studies underline the importance of usability, digital competence and organisational support in adopting digital tools, but they do not address customer portals as work tools. Consequently, the usability and usefulness of customer portals from the perspective of OHC professionals remain unexamined. This study examines the experiences of OH physicians, OH nurses, OH physiotherapists and OH psychologists regarding the usability and usefulness of customer portals.

The research questions are.

1. What is the usability and usefulness of customer portals as experienced by occupational health care professionals?
2. Which factors are associated with occupational health care professionals’ experiences of the usability and usefulness of customer portals?

## Materials and methods

### Research design

This sub-study was part of a broader project conducted by the Finnish Institute of Occupational Health, which examined the experiences of employee customers, employer customers, and OHC professionals regarding digital customer portals. This cross-sectional study examines OHC professionals’ experiences of the usability and usefulness of customer portals they use in their daily work.

### Participants, data collection and sample size

The study population consisted of OHC professionals. Data were collected through an online survey between 10 April and 27 May 2024. Contact persons appointed by professional unions and associations distributed the survey link via email, and one reminder was sent to encourage participation. No predefined inclusion or exclusion criteria were applied, as the survey link was sent through the member organisations representing the participating OHC professional groups. Participation was therefore open to all professionals within these organisations. All returned questionnaires contained sufficient information for analysis, and none needed to be excluded.

Because the link was distributed through contact persons, the exact number of professionals reached could not be determined. For the reporting purposes, the organisations’ total membership (N = 3200) was therefore used as an approximate frame population when calculating response rates.

In addition, a finite population sample size calculation was performed to illustrate the number of responses that would be required in a probability-based survey of this population. Using a 95% confidence level, a 5% margin of error and p = 0.5, the required sample size for a population of this size is approximately 341. This calculation does not apply to the present study, as the sample was obtained through voluntary participation and thus constitutes a non-probability sample.

Of the 3,200 members, 426 opened the link and 95 completed the survey, resulting in an overall response rate of 3%. When calculated from those who opened the link, the response rate was 22%. The realised sample (n = 95) reflects voluntary participation. While it is not a probability sample and does not aim at statistical representativeness, it was sufficient for the descriptive and non-parametric analyses used in this study.

### Consent to participate

Written consent was not specifically requested for this study. In Finland, completing a survey is generally considered an indication of consent to participate and to allow the use of collected data for research purposes. Each participant received a formal cover letter explaining the study background, voluntary and anonymous participation, and that submitting the questionnaire would be interpreted as informed consent. The survey also included a statement requesting permission to use responses for scientific research. The Ethical Board of the Finnish Institute of Occupational Health approved the consent procedure.

### Survey development

The questionnaire assessed the usability and usefulness of the customer portal. Usability was measured using 14 statements based on Nielsen’s^32^ five dimensions: learnability, efficiency, memorability, errors and satisfaction. Usefulness was measured using 15 statements derived from researchers’ expertise in OHC and Davis’s^33^ Technology Acceptance Model. Responses were recorded on a five-point Likert scale ranging from 1 (strongly disagree) to 5 (strongly agree). Prior to data collection, the survey was pre-tested by OHC specialists to ensure clarity and comprehensibility. The full questionnaire, originally developed in Finnish and translated into English for reporting purposes, is provided as a supplementary file (see Supplementary 1).

Although the questionnaire does not represent a previously validated instrument as a whole, the survey items were grounded in these widely used theoretical frameworks and underwent expert review prior to data collection, supporting their content validity. Internal consistency was excellent (Cronbach’s alpha 0.911 for usability and 0.920 for usefulness).

### Data analysis

Data analysis included descriptive statistics such as mean, standard deviation, skewness and kurtosis. Missing responses were excluded from analyses. Sum variables were formed from attitude statements, and internal consistency was assessed using Cronbach’s alpha (usability α = 0.911; usefulness α = 0.920). Statements were aligned in the same direction before forming sum variables, which were calculated only for respondents who answered all items. Associations between individual statements and sum variables were examined using Spearman’s rank correlation coefficient.

Relationships between respondents’ background factors and sum variables were analysed using non-parametric tests. Dichotomous variables were compared using the Mann–Whitney U test and multicategory variables using the Kruskal–Wallis H test. For Kruskal–Wallis tests, the effect size was reported as eta squared (η^2^), which describes the magnitude of differences between groups on a scale from 0 to 1, with interpretation thresholds of 0.01–<0.06 (small effect), 0.06–<0.14 (moderate effect), and ≥0.14 (large effect).^34,35^ For Mann–Whitney tests, the effect size r was used, which is based on the Z value and describes the difference between two groups on a scale from –1 to +1 (interpretation uses the absolute value).^36^ The interpretation thresholds for this scale are 0.1 (small effect), 0.3 (moderate effect), and 0.5 (large effect).^34^ Both effect sizes complement p-values by indicating the practical significance of the observed differences. Non-parametric methods were preferred because the sum variables were based on an ordinal Likert scale, even though normality tests did not indicate significant deviation from normality. For statistical testing, variables were dichotomised as follows: age (over 50 years/50 years or younger), gender (women/men), portal usage skills (good/poor or moderate) and satisfaction with the portal (satisfied/others). The threshold for statistical significance was set at *p*<0.05. Analyses were performed using SPSS Statistics software.

## Results

### Participants

Of the 95 OHC professionals who responded to the survey, the majority were OH nurses (n=38/95, 40 %) and OH physicians (29/95, 31 %). Most respondents were women (n=81/95, 85 %) and at least 50 years old (n=55/95, 58%). Nearly half of the respondents assessed their skill in using the customer portal as good (42/92, 46 %), while over half rated their skills as moderate (n=35/92, 38 %) or poor (n=15/92, 16 %). The background characteristics of the respondents are presented in Table 1.

**Table 1.** Respondents’ characteristics.

Characteristics		n	%
Profession (n = 95)	OH physician	29	31
	OH nurse	38	40
	OH physiotherapist	12	13
	OH psychologist	16	17
Age (n = 95)	Under 40 years	19	20
	40–49 years	21	22
	50–59 years	32	34
	60 years or more	23	24
Gender (n = 95)	Female	81	85
	Male	11	12
	Other/Don’t want to say	3	3
Customer portal usage skill (n = 92)	Poor	15	16
	Moderate	35	38
	Good	42	46
Work experience (n = 95)	Under 2–5 years	22	23
	6–10 years	16	17
	11–15 years	16	17
	16–20 years	21	22
	Over 20 years	20	21
Amount of employer customers (n = 94)	1	14	15
	2–10	12	13
	11–50	29	31
	51–100	19	20
	101–200	14	15
	Over 200	6	6
Amount of employee customers (n = 92)	Up to 600	15	16
	601–900	22	24
	901–1200	14	15
	1201–1500	9	10
	1501–1800	7	8
	Over1800	25	27
Satisfaction with the portal as a work tool (n = 95)	Completely dissatisfied	4	4
	Somewhat dissatisfied	9	10
	Neither dissatisfied nor satisfied	30	32
	Somewhat satisfied	37	39
	Completely satisfied	6	6
	No answer	9	10

### Usability of digital work tools (customer portals)

OHC professionals’ experiences of the usability of the customer portal were assessed using 14 statements. Appendix 1 illustrates the distribution of responses for these statements, and Table 2 presents the means and statistical indicators.

**Table 2.** Statistical indicators for statements measuring the usability of the occupational health care customer portal. The assessment is based on a five-point Likert scale (1 = strongly disagree, 5 = strongly agree).

Statements of usability	n	Mean	SD	Skewness	Kurtosis
It is easy to learn to use the portal.	90	3.78	1.03	-0.625	-0.168
The portal is easy to use.	88	3.73	1.06	-0.560	-0.413
I can easily find the information I need in the portal.	87	3.35	1.01	-0.257	-0.456
The way information is presented in the portal is clear and understandable.	87	3.36	1.07	-0.114	-0.671
The portal enables me to quickly accomplish what I want.	86	3.23	1.06	-0.300	-0.336
Using the portal is smooth even after a break.	87	3.60	1.05	-0.480	-0.290
Correcting errors made in the portal is easy.	87	2.67	0.95	-0.034	0.148
I experience only few usage errors in the portal.	87	3.46	0.95	-0.215	0.172
The portal’s functions are combined into a well-integrated whole.	86	3.02	1.07	0.011	-0.242
The text size in the portal is easy to read.	85	3.81	0.96	-0.611	0.275
The portal’s functions meet my expectations.	84	3.14	1.01	-0.294	-0.178
The user’s needs have been fully considered in the portal’s functions.	83	3.05	1.07	-0.282	-0.566
I do not fear unauthorised access to the portal’s data.	85	4.17	0.91	-0.529	-1.137
I do not need guidance for using the portal.	85	3.11	1.19	0.230	-0.923

Regarding usability the highest ratings were given to statements indicating that there is no fear of unauthorised access to portal data (mean 4.17), the text size is easy to read (3.81) and the portal is easy to learn to use (3.78). The response distributions for these statements were skewed towards the positive end of the scale (negative skewness: -0.529 to -0.625) and concentrated near the mean (kurtosis: -1.137 to 0.275). The lowest ratings were related to statements indicating that errors can be easily corrected (mean 2.67), the functions form a coherent whole (3.02) and the user’s needs have been fully considered (3.05). For these, the response distributions were more even (skewness close to 0: -0.282 to 0.011) and spread more widely across different response options (negative kurtosis: -0.566 to -0.242).

### Usefulness of digital work tools (customer portals)

OHC professionals’ experiences of the usefulness of the customer portal were assessed using 15 statements. The frequency distributions of responses are presented in Appendix 2 and the means and statistical indicators in Table 3.

**Table 3.** Statistical indicators for statements measuring the usefulness of the occupational health care customer portals. The assessment is based on a five-point Likert scale (1 = strongly disagree, 5 = strongly agree).

Statements of usefulness	n	Mean	SD	Skewness	Kurtosis
Using the portal makes my work easier.	89	3.83	1.01	-0.92	0.87
The information contained in the portal is always up to date.	87	3.55	0.91	-0.39	-0.25
Using the portal makes it easier to contact the customer.	87	3.66	1.13	-0.62	-0.37
Using the portal supports collaboration with the customer.	86	3.92	0.96	-0.89	0.72
Using the portal speeds up service delivery.	87	3.83	1.00	-0.71	0.11
Using the portal improves the quality of service.	86	3.92	0.95	-0.68	0.05
The portal helps me gain a better understanding of employees’ work ability.	88	3.75	1.20	-0.82	-0.15
The portal helps me identify those at risk of reduced work ability earlier.	88	3.75	1.29	-0.84	-0.36
The portal helps me gain a better overall picture of the workplace conditions.	87	3.36	1.32	-0.56	-0.81
The portal provides information on the impact of working conditions on health and work ability.	87	3.26	1.24	-0.41	-0.74
The portal provides information on workplace survey recommendations for supporting work ability.	87	3.08	1.19	-0.24	-0.69
The portal provides information on workplace survey recommendations for improving working conditions.	87	3.06	1.16	-0.34	-0.72
The portal enables me to monitor the implementation of recommendations given in workplace surveys.	87	2.63	1.15	0.02	-0.82
The portal provides information on the content of the occupational health care action plan.	87	2.90	1.29	-0.11	-1.05
The portal enables me to monitor the implementation of the occupational health care action plan.	87	2.82	1.21	-0.08	-0.93

Regarding usefulness, the highest ratings were given to statements indicating that the portal supports collaboration with the customer (mean 3.92), improves the quality of service (mean 3.92) and speeds up service delivery (mean 3.83). The response distributions for these statements were skewed towards the positive end of the scale (negative skewness: -0.68 to -0.89). In addition, the distributions were concentrated near the mean (positive kurtosis: 0.05 to 0.72). The lowest ratings were related to statements indicating that it is possible to monitor the implementation of workplace survey recommendations (mean 2.63), the implementation of the OHC action plan (mean 2.82) and the content of the action plan (mean 2.90). For these, the distributions were more even (skewness close to 0: -0.11 to 0.02) and spread more widely across different response options (kurtosis: -1.05 to -0.82).

### Factors associated with the usability and usefulness of the customer portal

Among the background factors, age, gender, work experience, and the categorised variables describing the number of customers were not associated with the assessments of usability or usefulness, and their effect sizes were very small or even 0. There was a statistically significant but moderate difference between professional groups regarding usefulness (p = 0.026, ε^2^ = 0.073), but not regarding usability (p = 0.060, ε^2^ = 0.051). In contrast, portal usage skills were associated with the experience of usability (p < 0.001, r = 0.498), indicating a moderate effect. The association was also observed with usefulness (p = 0.008, r = 0.280), but the effect was smaller. In addition, satisfaction with the portal as a tool was clearly associated with both usefulness (p < 0.001, r = 0.487) and the experience of usability (p < 0.001, r = 0.611). Professionals with good portal skills rated usability clearly higher (median 3.86 vs. 3.04), and those satisfied with the portal as a work tool gave substantially higher ratings for both usability (median 3.79 vs. 3.00) and usefulness (median 3.87 vs. 3.03) (Table 4).

**Table 4.** Non-parametric test results for differences in perceived usability and usefulness across respondent characteristic groups.

Characteristics	Usability					Usefulness				
	n	Median (Q1:Q3)	Test statistic	Effect size (η^2^/r)	*p*	n	Median (Q1:Q3)	Test statistic	Effect size (η^2^/r)	*p*
Profession*	90	​	H(df)=7.415 (3)	η^2^=0.051	0.06	​	​	H=9.250 (3)	η^2^=0.073	0.026
OH physician	26	3.39 (2.41:3.86)	​	​	​	26	3.04 (2.47:4.02)	​	​	​
OH nurse	38	3.71 (3.08:4.00)	​	​	​	37	3.60 (3.23:4.00)	​	​	​
OH physiotherapist	12	3.00 (2.88:3.45)	​	​	​	12	3.00 (2.87:3.38)	​	​	​
OH psychologist	14	3.36 (2.98:3.70)	​	​	​	14	3.67 (3.23:4.10)	​	​	​
Age **	90	​	U=931.000	r=0.055	0.61	​	​	U=949.500	r=0,017	0.871
Over 50 years	51	3.43 (2.93:3.86)	​	​	​	51	3.43 (2.93:4.07))	​	​	​
50 years or younger	39	3.50 (3.00:3.93)	​	​	​	38	3.47 (2.97:4.00)	​	​	​
Gender**	89	​	U=386.000	r=0.097	0.36	​	​	U=407.000	r=0.063	0.551
Female	77	3.43 (2.93:3.91)	​	​	​	76	3.47 (2.95:3.98)	​	​	​
Male	12	3.46 (2.89:3.61)	​	​	​	12	3.20 (2.30:4.00)	​	​	​
Portal usage skill**	89	​	U=414.000	r=0.498	<.001	​	​	U=648.500	r=0.280	0.008
Good	41	3.86 (3.36:4.36)	​	​	​	41	3.80 (3.10:4.07)	​	​	​
Moderate or poor	48	3.04 (2.80:3.55)	​	​	​	47	3.27 (2.87:3.60)	​	​	​
Work experience*	90	​	H(df)=1.994 (4)	η^2^=0	0.85	​	​	H=3.468 (4)	η^2^=0	0.628
Under 2–5 years	19	3.50 (2.93:3.93)	​	​	​	19	3.53 (3.00:3.93)	​	​	​
6–10 years	16	3.54 (2.84:3.88)	​	​	​	15	3.60 (2.87:4.07)	​	​	​
11–15 years	16	3.64 (3.00:3.98)	​	​	​	16	3.53 (3.30:4.15)	​	​	​
16–20 years	21	3.36 (2.46:3.82)	​	​	​	21	3.07 (2.73:3.97)	​	​	​
Over 20 years	18	3.43 (2.91:3.75)	​	​	​	18	3.37 (2.73:3.95)	​	​	​
Amount of employer clients*	82	​	H(df)=6.209 (5)	η^2^=0.016	0.40	​	​	H=4.958 (5)	ε^2^=0	0.549
1	6	3.43 (3.07:3.76)	​	​	​	6	3.17 (2.57:3.50)	​	​	​
2–10	11	3.64 (3.08:3.86)	​	​	​	10	3.43 (2.76:3.70)	​	​	​
11–50	27	3.50 (3.00:4.00)	​	​	​	27	3.53 (3.00:4.07)	​	​	​
51–100	18	3.61 (3.00:4.04)	​	​	​	18	3.50 (2.98:4.35)	​	​	​
101–200	14	3.00 (2.86:3.70)	​	​	​	14	3.57 (2.87:4.00)	​	​	​
Over 200	6	3.31 (2.36:3.96)	​	​	​	6	3.33 (2.47:3.88)	​	​	​
Amount of employee clients	80	​	H(df)=11.044 (5)	η^2^=0.082	0.14	​	​	H=6.806 (5)	η^2^=0.024	0.449
Up to 600	7	3.93 (3.79:4.00)	​	​	​	7	4.00 (3.00:4.27)	​	​	​
601–900	20	3.68 (3.02:3.93)	​	​	​	19	3.80 (3.07:4.29)	​	​	​
901–1200	14	3.36 (2.64:3.96)	​	​	​	14	3.80 (3.07:4.29)	​	​	​
1201–1500	8	3.57 (3.38:4.34)	​	​	​	8	3.80 (3.07:4.29)	​	​	​
1501–1800	7	3.62 (3.07:3.71)	​	​	​	7	3.80 (3.07:4.29)	​	​	​
Over 1800	24	3.00 (2.80:3.61)	​	​	​	24	3.80 (3.07:4.29)	​	​	​
Portal satisfaction as a work tool**	86	​	U=269.000	r=0.611	<.001	​	​	U=389.500	r=0.487	<.001
Satisfied	43	3.79 (3.43:4.36)	​	​	​	43	3.87 (3.40:4.20)	​	​	​
Others	43	3.00 (2.64:3.36)	​	​	​	42	3.03 (2.73:3.47)	​	​	​

*Note. Kruskall-Wallis H.

** Mann-Whitney U; OH = occupational health.

When examining the association of statements measuring usability and usefulness with the sum variables (Table 5), the strongest correlations with the usability sum variable were found in the statements *I can easily find the information I need in the customer portal* (correlation 0.786) and *The way information is presented in the customer portal is clear and understandable* (correlation 0.765). The strongest correlations with the usefulness sum variable were observed in the following statements: *The customer portal helps me gain a better overall picture of workplace conditions* (correlation 0.758), *The customer portal provides information on the impact of working conditions on employees’ health and work ability* (correlation 0.744), and *The customer portal provides information on recommendations for supporting employees’ work ability* (correlation 0.735).

**Table 5.** Correlation of statements measuring the usability and usefulness of the occupational health care customer portal with the composite variable.

Väittämä	n	ρ
Usability of the occupational health care customer portal		
It is easy to learn to use the portal.	90	0.677
The portal is easy to use.	88	0.718
I can easily find the information I need in the portal.	87	0.786
The way information is presented in the portal is clear and understandable.	87	0.765
The portal enables me to quickly accomplish what I want.	86	0.745
Using the portal is smooth even after a break.	87	0.721
Correcting errors made in the portal is easy.	87	0.429
I experience only few usage errors in the portal.	87	0.532
The portal’s functions are combined into a well-integrated whole.	86	0.592
The text size in the portal is easy to read.	85	0.653
The portal’s functions meet my expectations.	84	0.719
The user’s needs have been fully considered in the portal’s functions.	83	0.636
I do not fear unauthorised access to the portal’s data.	85	0.247
I do not need guidance for using the portal.	85	0.453
Usefulness of the occupational health care customer portal		
Using the portal makes my work easier.	89	0.566
The information contained in the portal is always up to date.	87	0.475
Using the portal makes it easier to contact the customer.	87	0.407
Using the portal supports collaboration with the customer.	86	0.620
Using the portal speeds up service delivery.	87	0.588
Using the portal improves the quality of service.	86	0.684
The portal helps me gain a better understanding of employees’ work ability.	88	0.671
The portal helps me identify those at risk of reduced work ability earlier.	88	0.574
The portal helps me gain a better overall picture of the workplace conditions.	87	0.758
The portal provides information on the impact of working conditions on health and work ability.	87	0.744
The portal provides information on workplace survey recommendations for supporting work ability.	87	0.735
The portal provides information on workplace survey recommendations for improving working conditions.	87	0.669
The portal enables me to monitor the implementation of recommendations given in workplace surveys.	87	0.643
The portal provides information on the content of the occupational health care action plan.	87	0.648
The portal enables me to monitor the implementation of the occupational health care action plan.	87	0.680

*Note.* All correlations are Spearman’s rank-order coefficients (ρ).

## Discussion

The study examined OHC professionals’ experiences of the usability and usefulness of digital work tools (customer portals). As this topic has been little studied in the context of OHC, the results provide valuable information for the development of portals. The identified development needs related to content, functionalities, and use may strengthen the role of portals in supporting professionals in their daily customer work.

Based on the survey, usability was generally assessed positively. Respondents considered the portals easy to use, easy to learn and clear. A positive experience was particularly reinforced by clear and accessible information. Perceived data security also emerged as a strength, supporting the use of the portal in confidential work situations and possibly partly explaining the positive attitude towards basic usability. In contrast, correcting errors, considering user needs and the coherence of functions received lower ratings. The results support the view that usability is not limited to technical functionality but also includes the user’s experience of functional integration.^32,37,38^ Previous studies show that system fluency promotes use, especially when it operates reliably and orientation is sufficient.^15,39–42^

Research also suggests that certain features of digital systems may make specific tasks more demanding for professionals. Automatically imported information can support single data entry and reduce manual work, but information may also become lengthy or repetitive if its structure does not align with practical work needs.^21–23^ When this occurs, identifying relevant content in customer portals may require more cognitive effort, which may also affect how well the system is perceived to respond to user needs.

OHC professionals’ assessments of the usefulness of the portal were also positive. In general, perceived usefulness promotes system adoption and long-term use.^40^ OHC customer portals were considered useful particularly in supporting collaboration, speeding up service delivery and maintaining contact with the customer. Portals can support information exchange and monitoring of agreed measures.^43^ According to our results, their current content does not appear to fully support OHC professionals’ work objectives. Some professionals felt that the portal does not provide a sufficient overall picture of the customer’s work ability and working conditions, nor does it allow monitoring of the implementation of workplace survey recommendations or the OHC action plan. The current information content of portals seems to primarily support the display of individual-level (employee-level) information. Examination of correlations and means of statements also suggests that although workplace-level information is considered important, its availability in the portal remains weaker. This is a clear shortcoming, as the effectiveness of OHC activities requires comprehensive and up-to-date information on workplace conditions.^44^ Similarly, previous studies have shown that systems that are easy to operate do not necessarily support professionals’ practical tasks or the formation of a comprehensive situational overview. This usability–usefulness gap has been identified across various professional digital tools.^24–26^ Our findings reflect this gap, as workplace-level information was experienced as insufficiently available in the portal.

Satisfaction with the portal was related to its perceived usability and usefulness, but variation was observed in the assessments. This variation may be explained by the fact that views and access rights are not necessarily uniform for all professionals. The results support previous findings that portals can streamline customer work when integrated into work processes,^15^ and their functionality and perceived usefulness strengthen commitment to use.^33^ However, it has been recognised that technical problems such as network connectivity issues can impair the experience of system fluency, increase workload, take time away from customer work and reduce willingness to use the system.^9,43,45,46^ In addition, mandatory use of the system and insufficient integration into clinical workflows weaken intentions to use.^20,37,46^

The aim of collaboration between OHC and workplaces is to prevent work-related illnesses and accidents, promote health and work ability and improve working conditions.^11,12^ Achieving these goals requires timely, comprehensive and traceable information on workplace conditions and employees’ situations.^29^ In addition to information, workplaces expect OHC to take a proactive approach and provide clear communication on managing workload factors in the work environment.^47–50^ Active participation in developing the work environment supports effective prevention of work-related diseases and occupational illnesses.^43,51^ The results showed that statements related to the employee level received clearly more positive ratings than those related to the workplace level. Thus, portals appear to support OHC professionals better in individual-level collaboration than in workplace-level collaboration.

However, customer portals can support collaboration goals if their content and functionality are developed to better meet the needs of workplace-related information management. The difference between professional groups in usefulness assessments and the observation that better user skills and greater satisfaction were associated with higher usability and usefulness sum variables suggest that customer portals do not serve all OHC professionals equally. On the other hand, although portals can support customer collaboration and speed up service, they also change interaction. As face-to-face contacts decrease, building trust, especially in new customer relationships, can be challenging, requiring careful planning of portal implementation and clear practices for managing customer relationships.^52^ In addition, complex situations and sensitive issues often still require face-to-face contact to ensure adequate understanding and support^31^ It is also important to note that all types of digital customer services require professionals to have experience in face-to-face customer work, as digital means alone may not provide sufficient understanding in every situation.^30^

System developers consider professional involvement in development work important, but it often remains limited to system testing and does not extend to specification or design.^53^ Feedback collection should be continuous.^54,55^ Customer portals should be developed to integrate information from different sources and provide a situational overview of factors affecting work ability, health and safety for all OHC professionals responsible for customers. In addition, they should enable monitoring of agreed measures to assess the effectiveness of activities. Another noteworthy finding was the large proportion of neutral responses in some statements, suggesting that some professionals cannot form a clear assessment, for example, of error management and functional coherence. This should also be considered in the development of customer portals.

In summary, the perceived usability and usefulness of the OHC customer portal are particularly related to how easily professionals can find the information they need and how clearly the information is presented. Overall, the study shows that customer portals have the potential to support the goals of OHC, but their effective use requires more comprehensive alignment of technology, operations and professionals’ needs.

Sittig and Singh’s^10^ sociotechnical model examines the successful use of health information systems as an interaction between technology, people, processes and the organisation. Future research should examine the use of digital tools in OHC more strongly from a sociotechnical perspective, which would increase understanding of their impact on professionals’ everyday work and collaboration with workplaces and their employees.

### Strengths and limitations

This study provides novel insights into OHC professionals’ experiences of the usability and usefulness of digital work tools, an area that has been scarcely investigated. The timeliness of the study is a strength, as digitalisation is rapidly transforming OHC practices and creating new demands for effective digital work tools. Another strength is that the study applied well-established theoretical frameworks, such as Nielsen’s^32^ usability dimensions and Davis’s^33^ Technology Acceptance Model, which provided a robust basis for the measurement of key concepts and ensured comparability with previous research. The internal consistency of the sum variables was excellent (Cronbach’s alpha >0.90), which confirms that the scales used were reliable and coherent. In addition, the survey was carefully designed and pre-tested with experts, which improved clarity and relevance of the questions and supports the credibility of the data. This study fills a research gap by highlighting issues that are important for OH digital work tools’ development and implementation.

However, the study also has limitations. The relatively small sample size and the skewed gender distribution (predominantly female respondents) restrict the generalisability of the results. The low response rate may indicate a risk of selection bias, as those more interested in digital tools might have been more likely to participate. Additionally, self-reported assessments may also be subject to response bias, although they are often the only feasible way to capture subjective experiences. Moreover, the study did not identify or take into account potential differences between digital work tools, which may influence user experience. This also limits the interpretability and reproducibility of the usability assessments, because it is unclear whether the reported experiences represent general views or are connected to specific customer portals. Therefore, caution should be exercised when generalising the findings to all OHC professionals or their digital work tools.

In addition, the study applied only one of the core constructs of the Technology Acceptance Model (TAM), namely perceived usefulness. Our findings may also reflect determinants included in later acceptance models, such as TAM2 and the Unified Theory of Acceptance and Use of Technology (UTAUT), which incorporate social influence and facilitating conditions as additional predictors of technology use.^56^ Differences between professional groups or variation in access rights, as well as challenges related to workflow integration, may be partly explained by these broader determinants. For example, if an OHC organisation does not mandate the use of the customer portal, professionals may be less inclined to participate in related surveys, contributing to the low response rate. Likewise, variation between professional groups may reflect differences in training practices and the clarity of portal related responsibilities. Although these mechanisms were not assessed directly, recognising them provides a useful theoretical frame for interpreting variation in user experiences and represents an important direction for future research.

## Conclusions

Digital work tools, such as customer portals, are an established part of OHC professionals’ work, but their potential to promote employees’ work ability and improve workplace conditions is not yet fully realised. The results indicate that portals support employee-level information management more effectively than they enable forming an overall picture of the workplace situation or monitoring the implementation of agreed measures. Development should therefore focus on strengthening portal content and functionalities, taking into account OH services directed at both employees and workplaces. In addition, it is essential to ensure that the functions meet the needs of different professional groups and accommodate variations in user skills so that portals serve as effective tools for all OHC professionals.

## Supplemental material

Supplemental material - Digital work tools in occupational health care: A cross-sectional survey of usability and usefulness among occupational health professionals in Finland Supplemental material for Digital work tools in occupational health care: A cross-sectional survey of usability and usefulness among occupational health professionals in Finland by Sari Nissinen, Pauliina Toivio and Erja Sormunen in Digital Health.

## Figures and Tables

**Appendix 1.**
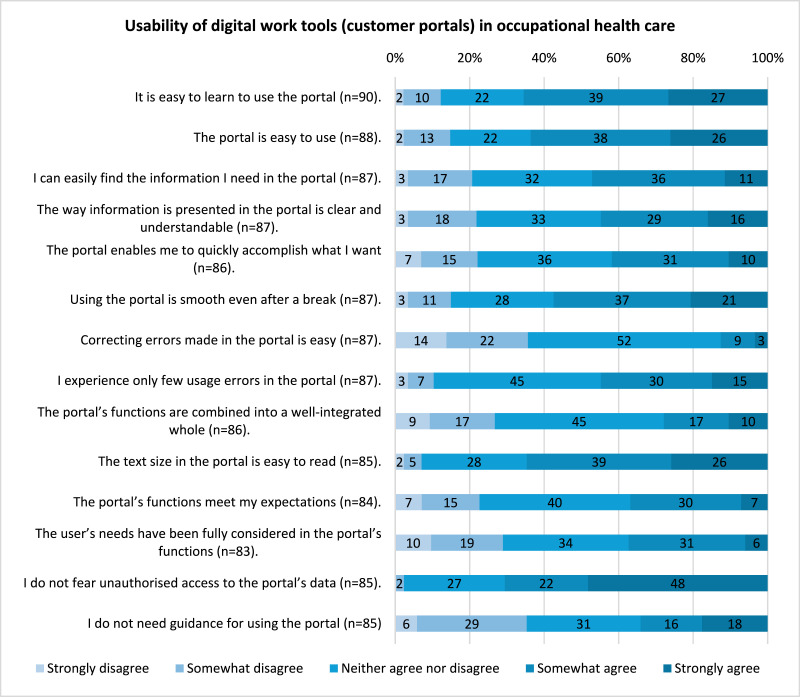
Respondents’ attitude statements regarding the perceived usability of digital work tools (customer portals) in occupational health care.

**Appendix 2.**
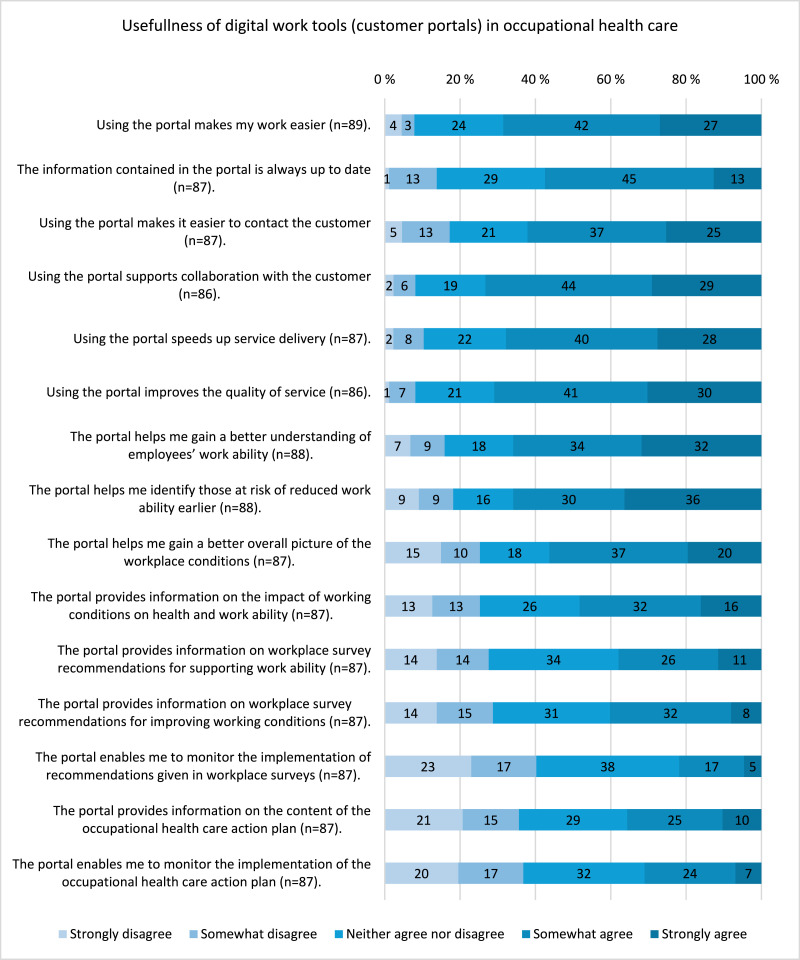
Respondents’ attitude statements regarding the perceived usefulness of digital work tools (customer portals) in occupational health care.

## Data Availability

The datasets generated and analyzed during the current study are not publicly available due to privacy restrictions but are available from the corresponding author on reasonable request.*
