# Supplementary material for: Digital work tools in occupational health care: A cross-sectional survey of usability and usefulness among occupational health professionals in Finland
Source: Digit Health. 2026 May 12;12:20552076261450324. doi: 10.1177/20552076261450324 (PMC13172705; doi:10.1177/20552076261450324)
Supplement: Supplemental material - Digital work tools in occupational health care: A cross-sectional survey of usability and usefulness among occupational health professionals in Finland [file sj-pdf-1-dhj-10.1177_20552076261450324.pdf]

Supplementary 1.
Questionnaire content **(Translation from Finnish)**

A customer portal refers to digital work tools (customer portals) that you use in your work with employee and employer customers.

1. What is your age? (please provide the nearest whole number) ____

2. What is your gender?
- Female
- Male
- Other or prefer not to say

3. What is your profession?
- Chief physician, head or responsible occupational health physician
- Occupational health physician
- General practitioner working in occupational health care
- Head or responsible occupational health nurse
- Occupational health nurse
- Head or responsible occupational physiotherapist
- Occupational physiotherapist
- Head or responsible occupational health psychologist
- Occupational health psychologist
- Social work specialist
- Customer relationship manager
- Service manager
- Other, please specify: ______

4. How many years have you worked in occupational health care? (please round to the nearest whole number)

5. How many workplaces are under your customer responsibility?
- None
- 1
- 2–10
- 11–50
- 51–100
- 101–200
- More than 200

6. How many employee clients are under your customer responsibility?
- Up to 300
- 301–600
- 601–900
- 901–1200
- 1201–1500
- 1501–1800
- More than 1800

7. How would you assess your skills in using the occupational health customer portal?
- Poor
- Moderate
- Good

8. How satisfied are you overall with the use of the occupational health customer portal in supporting your clients’ work ability and improving working conditions?
- Completely dissatisfied
- Quite dissatisfied
- Neither satisfied nor dissatisfied
- Quite satisfied
- Completely satisfied

9. Please indicate your level of agreement with the following statements about the usability of the occupational health customer portal (Response options: Strongly disagree, Somewhat disagree, Neither agree nor disagree, Somewhat agree, Agree, Strongly agree)

- Learning to use the customer portal is easy.
- The customer portal is easy to use.
- I can easily find the information I need in the customer portal.
- The way information is presented in the customer portal is clear and understandable.
- The customer portal allows me to quickly accomplish what I want.
- I can use the customer portal smoothly even after a break (e.g., vacation or other longer pause).
- Correcting mistakes (such as changing selections or fixing incorrect entries) is easy.
- I experience very few errors when using the customer portal.
- The different functions in the customer portal are well integrated into a coherent whole.
- The text size in the customer portal is easy to read.
- The functions of the customer portal meet my expectations.
- It is evident that user needs have been fully considered in the portal's functions.
- I am afraid that unauthorized persons might access information in the customer portal I use.
- I would like guidance on how to use the customer portal.

10. Please indicate your level of agreement with the following statements about the usefulness of the occupational health customer portal (Response options: Strongly disagree, Somewhat disagree, Neither agree nor disagree, Somewhat agree, Agree, Strongly agree)

- Using the portal makes my work easier.
- The information contained in the portal is always up to date.
- Using the portal makes it easier to contact the customer.
- Using the portal supports collaboration with the customer.
- Using the portal speeds up service delivery.
- Using the portal improves the quality of service.
- The portal helps me gain a better understanding of employees' work ability.
- The portal helps me identify those at risk of reduced work ability earlier.
- The portal helps me gain a better overall picture of the workplace conditions.
- The portal provides information on the impact of working conditions on health and work ability.
- The portal provides information on workplace survey recommendations for supporting work ability.
- The portal provides information on workplace survey recommendations for improving working conditions.
- The portal enables me to monitor the implementation of recommendations given in workplace surveys.
- The portal provides information on the content of the occupational health care action plan.
- The portal enables me to monitor the implementation of the occupational health care action plan.
